# Viral and cellular SOS-regulated motor proteins: dsDNA translocation mechanisms with divergent functions

**DOI:** 10.1186/2045-3701-4-31

**Published:** 2014-06-25

**Authors:** Annie Wolfe, Kara Phipps, Tao Weitao

**Affiliations:** 1 Biology Department, College of Science and Mathematics, Southwest Baptist University, 1600 University Ave, Bolivar, Missouri 65613, USA

**Keywords:** AAA+ proteins, DNA translocases, DNA repair, Replication, Recombination, SOS response, Bacteria, Phages

## Abstract

DNA damage attacks on bacterial cells have been known to activate the SOS response, a transcriptional response affecting chromosome replication, DNA recombination and repair, cell division and prophage induction. All these functions require double-stranded (ds) DNA translocation by ASCE hexameric motors. This review seeks to delineate the structural and functional characteristics of the SOS response and the SOS-regulated DNA translocases FtsK and RuvB with the phi29 bacteriophage packaging motor gp16 ATPase as a prototype to study bacterial motors. While gp16 ATPase, cellular FtsK and RuvB are similarly comprised of hexameric rings encircling dsDNA and functioning as ATP-driven DNA translocases, they utilize different mechanisms to accomplish separate functions, suggesting a convergent evolution of these motors. The gp16 ATPase and FtsK use a novel revolution mechanism, generating a power stroke between subunits through an entropy-DNA affinity switch and pushing dsDNA inward without rotation of DNA and the motor, whereas RuvB seems to employ a rotation mechanism that remains to be further characterized. While FtsK and RuvB perform essential tasks during the SOS response, their roles may be far more significant as SOS response is involved in antibiotic-inducible bacterial vesiculation and biofilm formation as well as the perspective of the bacteria-cancer evolutionary interaction.

## Introduction

**Figure 1:**
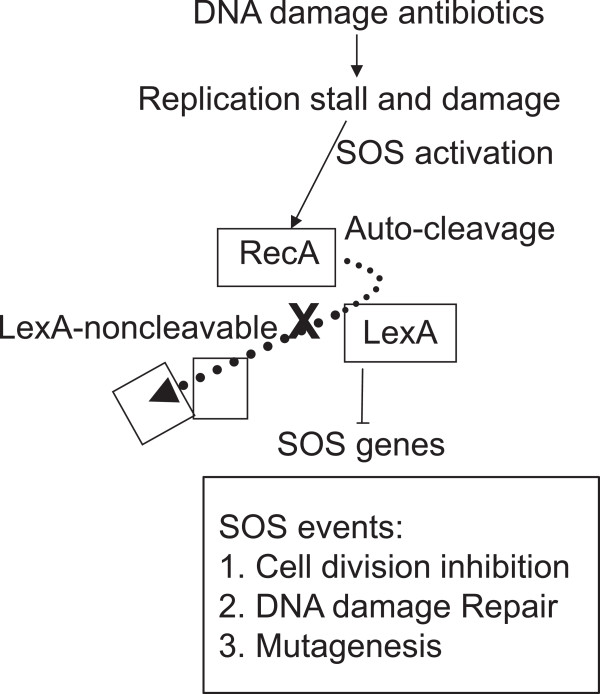
**The bacterial SOS machinery.** The SOS machinery is operated by the interplay of two key regulators, an SOS repressor LexA and an inducer RecA. RecA responds to DNA damage by binding to ssDNA, which triggers LexA autocleavage. The LexA repressor dissociates from the SOS boxes in order to derepress and induce transcription of the SOS regulon. These genes work to either repair or bypass the lesions of DNA damage.

**Figure 2:**
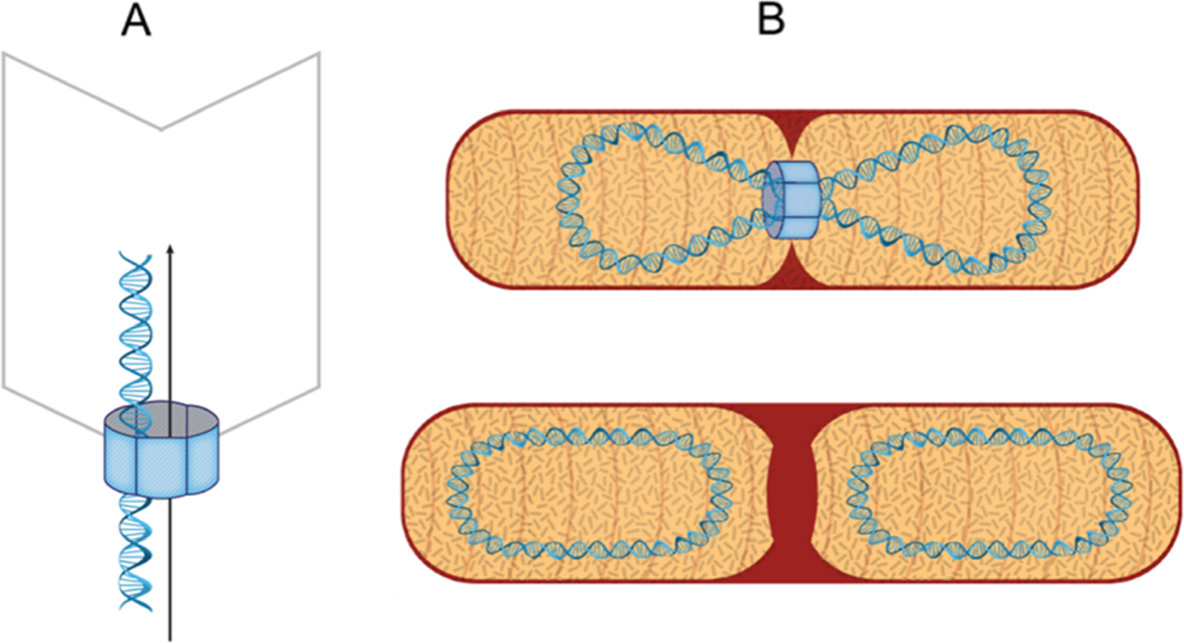
**Two types of ASCE hexameric dsDNA motors. (A)** Unidirectional motors represented by phage φ29 gp16 using a revolution mechanism with a power stroke between subunits through an entropy-DNA affinity switch to push dsDNA into the page head without rotation of DNA and the motor. **(B)** Bidirectional motors represented by bacterial FtsK employing the similar revolution mechanism. (Created by artist Grant Cochran).

Bacterial chromosomes and phages share a similar cycle of life: genome replication, packaging and segregation. When encountering stress such as DNA damage, the host cells launch SOS response (Figure 1) [1,2], in which the host cell adjusts to accommodate DNA damage. Chromosome stability and consequently, phages, are threatened, as the host cell delays cell division so that DNA damage can be fairly repaired. Phages multiply through viral genome replication, DNA packaging and assembly. One function essential in either the normal or the stressed conditions is translocation of double-stranded (ds) DNA, by which phage DNA is transported into the prohead (Figure 2A), chromosomes are translocated into daughter cells (Figure 2B), and damaged DNA is repaired (Figure 1). This function is carried out by dsDNA translocases that belong to the additional strand catalytic E (ASCE) superfamily, including the ATPases associated with a variety of cellular activities (AAA+) [3] and the FtsK-HerA superfamily [4]. This review attempts to summarize our understanding of the SOS regulated dsDNA translocases using a phi29 packaging motor as a prototype.

## A novel hexameric prototype of the bacteriophage motor proteins

DNA packaging and delivery machines in tailed dsDNA bacteriophages have been used as models for studying DNA motors (Figure 2A) [5], since these viruses have complex assembly pathways [6] that are representative of some cellular processes. Of these phages, the bacillus phage phi (φ) 29 was first discovered to have a portal head–tail connector in an icosahedral shell [7]. Located in a pentavalent site in the capsid, the portal fits within this opening and is composed of a connector channel with a 35 Å-diameter size at the narrowest part through which phage DNA is translocated during packaging. More interestingly, the gp16 ATPase of phi29 packaging motor, belonging to the ASCE superfamily, has drawn great attention. This class of nanomotors facilitates a wide range of functions including DNA damage repair, replication, recombination, chromosome segregation, DNA/RNA transportation, membrane sorting, and cellular reorganization [8,9]. This motor operates by a revolving mechanism without rotation in analogy to the Earth revolving around the sun, free of friction, coiling, and torque [10]. This mechanism has been discovered in DNA translocation motors of viruses, bacteria, and eukaryotic cells.

**Figure 3:**
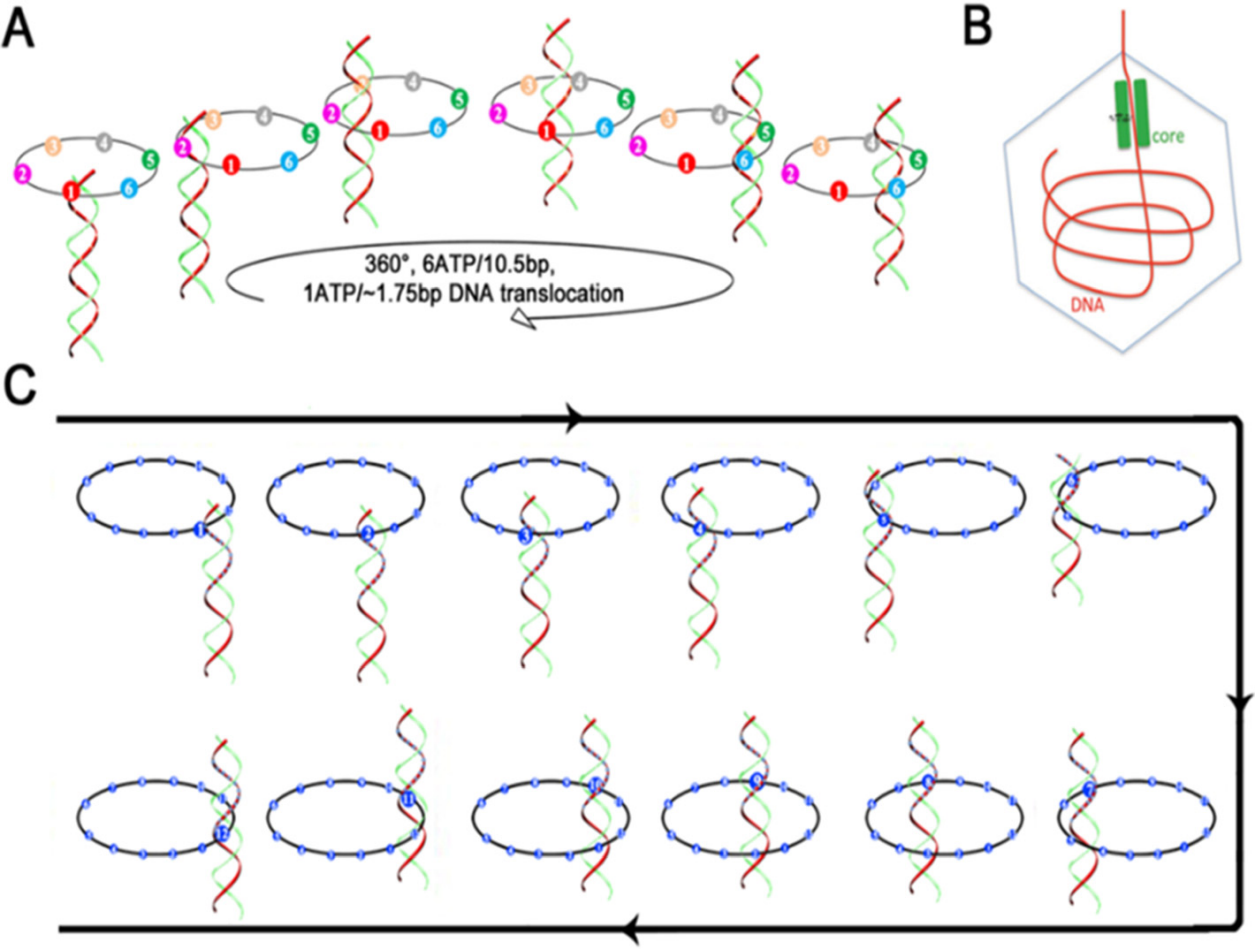
**The sequential revolution in translocating dsDNA. (A)** The φ29 DNA packaging motor is made of a hexameric ATPase gp16, a hexameric pRNA ring [11] and a dodecameric gp10 connector [12], which form three-coaxial rings [12]. During the viral DNA packaging, ATP shifts one subunit of ATPase gp16 toward a conformation with low entropy but high affinity for dsDNA, which is reversed once the ATP is hydrolyzed, causing a power stroke that pushes the dsDNA toward the adjacent subunit around the diameter of the ATPase tunnel wall. Six ATPs complete a cycle, with one ATP hydrolyzed per step, to achieve one helical turn of 360º (10.5 bp). Rotation of either the DNA or the hexameric ring is unlikely. **(B)** Diagram of CryoEM results showing the position of dsDNA in the channel wall of bacteriophage T7 DNA packaging motor. **(C)** The dsDNA revolving along the 12 subunits. (Adopted from reference [24] with the permission from the publisher).

The phi29 dsDNA packaging motor is made of a hexameric ATPase gp16, a hexameric pRNA ring [11] and a dodecameric gp10 connector [12]. The components form the three-coaxial rings through which dsDNA is translocated into the procapsid [12] (Figure 3) by a novel mechanism of revolution rather than rotation. The dodecameric gp10 connector of the motor is composed of 12 encircling subunits of gp10, forming a central channel, through which dsDNA is translocated [13,14]. The motor connector channel processes elastic properties and heterogeneous stiffness that prevent DNA leakage during translocation [15]. During viral DNA packaging, ATPase gp16 is stimulated by ATP binding to its subunit to adapt a conformational entropy with high affinity for dsDNA. When ATP is hydrolyzed, however, the ATPase switches to a different conformational entropy with lower affinity to dsDNA so that dsDNA leaves the subunit and moves to the next lower entropy-high affinity subunit by a power stroke (Figure 3). Six ATPs are consumed along the hexameric ring in one cycle, translocating the dsDNA one helical turn of 360° at 1.75 bp per ATP [16,17]. Because the DNA is actually revolving unidirectionally along the hexameric tunnel wall, it is unlikely for the DNA or the hexameric ring to rotate. This model is well supported by multiple lines of evidence from phi29 [16-21] and T4 DNA packaging motors [22] as well as bacterial FtsK [23], such as dsDNA affinity binding properties, observed cooperative and sequential subunit actions, predicted ring sizes, proper DNA binding orientation and subunit angles.

## The SOS response in bacteria

Unlike bacteriophages, bacterial populations have the capacity to launch an emergency response to environmental threats. This response is named after the international telegraph distress signal termed “SOS”. Early observations of irradiated phages and host bacterial cells as reviewed previously prompted the SOS response hypothesis [25]. When UV-irradiated phage λ was plated on irradiated *Escherichia coli* cells*,* reactivation of the irradiated phage increased significantly [26] in a phenomenon termed Weigle reactivation [1]. Additionally, when *E. coli* lysogens carrying prophage λ on the host chromosome were UV-irradiated, prophage induction was stimulated, resulting in host lysis and phage release [27-30]. The UV-irradiated *E. coli* cells also became filamentous indicating cell division inhibition. These radiation events of division arrest, prophage induction and UV-induced mutation, were related as suggested [31], which led Miroslav Radman to conclude that irradiated *E. coli* undergoes DNA damage repair through SOS response [1,2].

The SOS machinery is operated by interplay between two key regulatory proteins, an SOS repressor LexA (locus for X-ray sensitivity A [32]) and an inducer RecA (recombinase A), which alternatively turns SOS on or off (Figure 1A) [33] as reviewed recently [34]. In the absence of single-stranded DNA (ssDNA, which is a DNA damage signal), LexA represses expression of at least 43 SOS genes mostly involved in DNA damage repair in *E. coli*[35,36]. LexA autoregulates its expression by binding to its own boxes [37], thereby minimizing excessive states of LexA and increasing sensitivity to the SOS signal. In response to DNA damage signals, coprotease RecA, becomes activated and assumes a filament that binds to ssDNA [38,39]. The ssDNA-RecA coprotease triggers subsequent LexA autocleavage activity occurring between residues Ala84 and Gly85 [33]. The self-cleaved LexA repressor dissociates from its binding sites (SOS boxes) upstream of the SOS genes to derepress and induce SOS genes that act to repair or bypass DNA damage. The activity of RecA coprotease then declines, followed by dimerization of LexA which binds to the SOS boxes and represses the SOS gene expression [40].

## Bacterial dsDNA hexameric translocases under SOS control

The dsDNA translocases of the ASCE DNA motor proteins are critical to DNA repair, replication, recombination, chromosome segregation, DNA/RNA transportation, membrane sorting, cellular reorganization, and many other processes [8,9]. As observed in *E. coli*, the FtsK family of the ASCE protein family transports DNA and separates intertwined chromosomes during cell division (Figure 2B) [4], while the SpoIIIE family [41] translocates DNA from a mother cell into the pre-spore during sporulation of *Bacillus subtilis*[42]. Both FtsK and SpoIIIE DNA transportation systems rely on the assembly of a hexameric machine. Besides, functioning in a rotational fashion, TrwB transports DNA during bacterial conjugation [43,44]; replicative DNA helicase DnaB [45] unwinds dsDNA in the front of the replication fork to provide ssDNA templates for the DNA polymerase III holoenzyme [46,47]; and RuvB translocates dsDNA in an ATP hydrolysis-dependent manner during recombination [48]. These DNA motor proteins maintain routine functions of life; but a few, such as FtsK and RuvB, are induced during the SOS response to fulfill special tasks. These two proteins are reviewed below.

## FtsK

### Discovery of SOS-regulated *ftsK*

The *ftsK* gene was discovered by mutations in an *E. coli* cell division gene that rendered a temperature-sensitive late-stage arrest in division without affecting chromosome replication or segregation [49,50]. *ftsK* expression increases during SOS response [51]. The first of *ftsK*’s two promoters is situated within the *lrp* (global response regulatory gene) reading frame and is dispensable. The essential, second promoter corresponds to *dinH*, which previously was characterized as an SOS promoter [52]. The FtsK protein is a 147-kDa polypeptide. Its N-terminal domain (FtsK_N_) displays predicted membrane-spanning regions. The C-terminal domain (FtsK_C_), is a member of ASCE superfamily [4] with a nucleotide-binding consensus sequence [49]. FtsK bears extensive homology with bacterial proteins involved in DNA transfer, such as SpoIIIE of *B. subtilis*[49,50].

### Couple of chromosome segregation with cell division

FtsK couples chromosome segregation with cell division at the bacterial septum (Figure 2B) [53]. In the presence of a chromosome dimer, FtsK_C_ is brought to the Xer-*dif* nucleoprotein complex [54], which resolves the dimer to the monomers [55] through Xer site-specific recombination by two recombinases, XerC and XerD acting on a 28-bp recombination site on chromosome, *dif*[56-59]. FtsK_50C_, a truncated FtsK derivative that contains an intact C-terminal domain, is a DNA motor protein. Functioning as a DNA translocase and forming a ring-shaped multimer on a DNA template, it activates resolution of a chromosome dimer by switching the catalytic state of the XerC and XerD recombinases [60] on using ATP hydrolysis [61]. XerD generates Holliday junctions by creating a pair of strand exchanges and XerC resolves this structure through the reaction between directly repeated *dif* sites in circular DNA [60]. FtsK_C_ ATPase activity directly activates Xer recombination at *dif* before Holliday junction formation [61]. The terminal catenation of replicated chromosomes are thereby separated or decatenated to leave the septal region free of DNA before completion of cell division. FtsK may act directionally to ensure this separation (decatenation) directional action [62,63]. This premise is aligned with the decatenation process *in vitro* by using combination of the FtsK-XerCD recombination machinery that facilitates synapsis of *dif* during FtsK translocation along DNA and resolves chromosomal dimers to monomers [64]. The resolution is thought to be mediated by FtsK, which translocates chromosomal DNA through the closing septum in a DNA-sequence independent manner [65].

### Mechanism of DNA translocation

**Figure 4:**
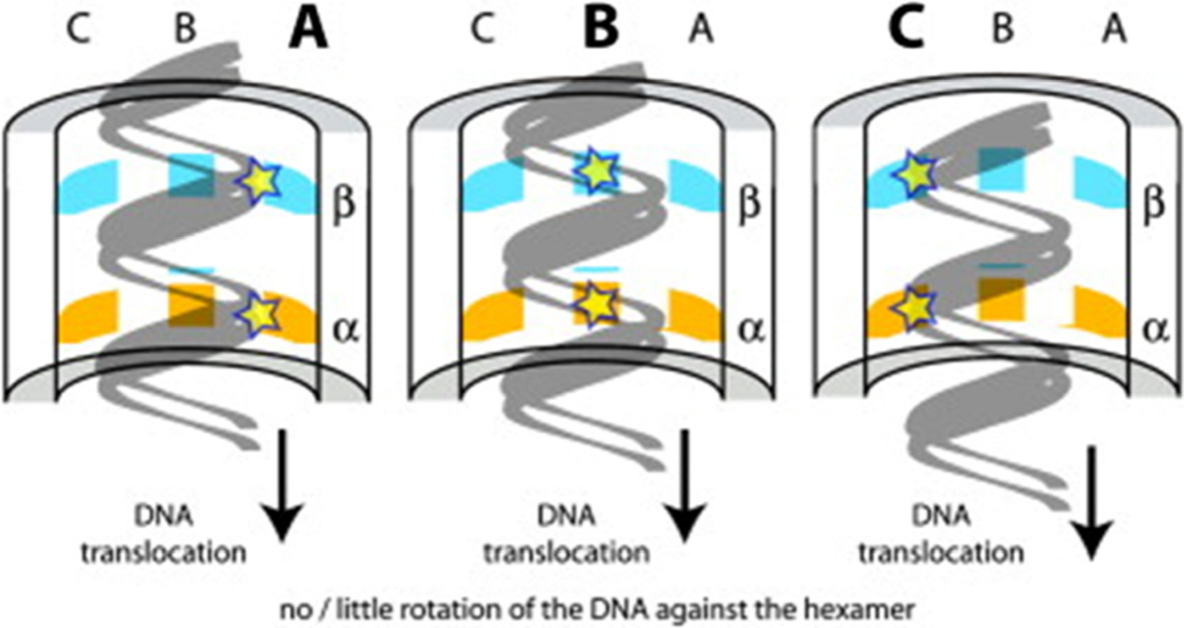
**A revolution model of DNA translocation by hexameric FtsK.** FtsK may employ a revolution mechanism to transport DNA without rotation. The hinged movement between α and β domains of PaFtsK_C_ ensures a continuous translocation of DNA through the hexameric FtsK_C_ ring, by which the α domain translocates the DNA backbone, and then releases as the β domain binds and moves the helix within the same patch. Stars, indicate the active subunit of each power stroke. (Adopted from reference [23] with the permission from the publisher).

FtsK translocates chromosomal DNA from the septum at cell division in a fascinating process (Figure 4), given FtsK’s versatility in translocation of DNA, control of the directionality, and self-anchorage to the DNA substrate [41]. FtsK is responsible for bidirectional dsDNA translocation [66] and may employ a revolution mechanism to transport DNA as indicated by the structural study (Figure 4) [23]. Specifically, FtsK of *E. coli* (EcFtsK) is a multi-domain protein consisting of a 600-amino acid linker, FtsK_C_ (α, β and γ), and FtsK_N_[60,67,68]. The ATP-dependent ability of EcFtsK to move on DNA molecules *in vitro* suggests that it is a DNA motor protein [23]. As EcFtsK’s long linker complicated structural studies of the motor mechanism, the C-terminal domain of *Pseudomonas aeruginosa* (PaFtsK_C_) was adopted for further investigations [23]. PaFtsK_C_ structural studies indicate a RecA-like core and a ring-like hexamer with DNA-dependent formation through which DNA passes. The α and β domains of FtsK_C_ make up the DNA translocase and γ interacts with XerD [23]. From these data, a “rotary inchworm” mechanism of dsDNA translocation similar to the revolving mechanism was proposed (Figure 4) [23,69]. Hexameric FtsK_C_ translocates DNA through its central channel where protein-DNA contacts involve one or two monomers, which undergo a catalytic cycle, translocating DNA without evident rotation as DNA binds the next subunit following the second subunit’s catalysis [23]. A rotation mechanism is unlikely, as predicted by the PaFtsK_C_ hexameric ring diameter being greater than that of dsDNA, suggesting a revolution mechanism analogous to the phi29 motor (Figure 3).

The revolving mechanism exercises hinged movement between the α and β domains of PaFtsK_C_ to translocate dsDNA (Figure 4) [23]. The hexameric ring holds dsDNA, with one functional subunit contacting the DNA at a time. The functional subunit monomer experiences an ATP catalytic cycle and translocates DNA through the channel by the hinged movement of the α and β domains (Figure 4) [23]. In particular, the α domain drags the DNA backbone to translocate 1.6 base pairs of the helix per ATP before releasing. The β domain subsequently binds to the next location of the helix within the same patch of the DNA and moves it. This action carries the DNA backbone to the next functional subunit inside the same ring by a sequential hand-off mechanism without rotation of the protein ring against the DNA [23] so that one functional subunit of the hexameric ring contacts the dsDNA at a time. It performs the same exercise of DNA-binding, a catalytic cycle and translocation. This DNA translocation cycle is facilitated by the interaction between helical structure of DNA and the functional subunit of the hexameric ring [23]. Furthermore, this cycle of DNA translocation may follow a sequential escort mechanism in which multiple α and/or β domains drag and release the DNA strand per catalytic step before changing hands with the adjacent subunits [70].

## RuvB

**Figure 5:**
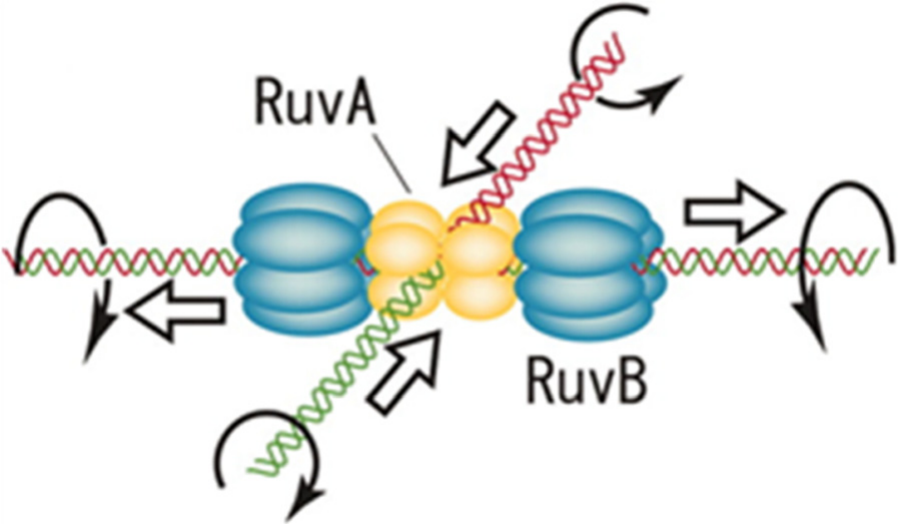
**Branch migration of RuvA-RuvB in solution.** The four monomers of RuvA combine around a central pen to accommodate the square planar configuration of the Holliday junction in which the four DNA duplex arms attach to grooves on the concave surface of RuvA. Through ATP hydrolysis, the two hexameric RuvB rings encircle and translocate the dsDNA arms. Curved arrows indicate rotation of DNA while the thick arrows indicate translocation of dsDNA through the junction. DNA rotation during Holliday junction branch occurs at a V(max) of 1.6 revolutions per second, or 8.3 bp per second. (Adopted from reference [76] with the permission from the publisher).

RuvA, RuvB, and RuvC, are three proteins that play important roles in processing Holliday junctions formed in the late stage of homologous recombination of prokaryotes (Figure 5) [48,71,72]. The genes for RuvA and RuvB are part of a LexA regulated SOS regulon [73]. RuvB has been classified as a member of the AAA+ ATPase superfamily, based on structural analysis [74,75].

### Discovery of SOS-regulated ruvAB operon

The *ruvA* and *ruvB* genes were discovered by mutations that rendered the *E. coli* cells sensitive to UV irradiation [77]. After exposure to UV light, the *ruvA* and *ruvB* mutants were found defective in cell division, resulting in nonseptate multinucleated cells [77]. This suggests an inability of these mutants for recombination and repair of UV damage, as well as involvement of the SOS response [78], In fact, the SOS boxes were found near a promoter of an operon containing two open reading frames for RuvA and RuvB [73,79] but not for RuvC, located immediately upstream of the *ruvAB* operon [80]. Furthermore, the *ruvAB* operon was induced by DNA damage [81] through LexA derepression [79] whereas *ruvC* was not [80,82].

### Interaction of RuvABC with Holliday Junctions

Holliday junctions are generated by RecA but processed by RuvAB and resolved by RuvC, with RuvB as an ATP-driven motor for branch migration (Figure 5) [83]. Asymmetric assembly of the RuvAB-branch migration complex was observed, in which RuvAB pushes DNA through the hexameric rings of RuvB and promotes branch migration [84]. Electron microscopy of the tripartite RuvAB-Holliday junction complex showed that RuvA binds to the crossover while RuvB forms two hexameric rings encompassing dsDNA on each side, so that the Holliday junction adopts a square-planar structure (Figure 5) [85]. Both proteins bind Holliday junctions, but RuvA loads RuvB onto the junctions [86]. Loading is mediated by RuvA targeting one hexameric RuvB ring to one arm of the DNA complex [86]; the DNA then can be pushed through the RuvB ring and unwound [87]. During branch migration, RuvC scans the region for cleavage sites during RuvAB-mediated branch migration, dissociates RuvA, and eventually cleaves the junction [88]. Based on a finding that RuvAB doesn’t necessarily impact the site specificity of RuvC-dependent cleavage, a model was proposed that the RuvABC resolvasome acts at the RuvC consensus cleavage sequence signaled by RecA through a Holliday junction formation [89]. Conclusively, RecA and the related proteins initiate formation of the Holliday junction from the lesion of DNA damage, while RuvAB catalyzes branch migration and recycles RecA [90] at the expense of ATP, and RuvC recycles RuvAB and resolves the junction.

### DNA translocation mechanism by RuvB

RuvB forms two hexameric rings, through which dsDNA is translocated in an ATP hydrolysis-dependent manner (Figure 5) [48,72]. EM revealed that RuvB’s two hexameric rings are arranged in a bipolar manner with the large ends faced inward enabling DNA to exit through the small ends [91]. The exact mechanism was later elucidated by a proposed atomic model for the RuvA–RuvB–Holliday junction complex, in which RuvB pumps in and out DNA duplex arms without segmental unwinding [92]. This modeled mechanism, derived from EM images of the ternary RuvA-B complex [93], seems different from the revolution mechanism of phi29 gp16 as described earlier (Figure 3). How RuvB rotates dsDNA still remains elusive, but a mechanochemical-coupling mechanism was proposed that two subunits of RuvB hexameric rings bind dsDNA and hydrolyze ATP to generate a power stroke and rotate DNA in a DNA binding-ATP hydrolysis step that relays along the ring [94]. Such a rotation was observed in an observational nanobead system in which one end of the cruciform DNA was fixed onto a glass bead surface [76]. Real-time observations suggest DNA rotation during Holliday junction branch migration at 1.6 revolutions per second (Figure 5) [76]. Since rotation of dsDNA in chromosome causes the topological stress and extra ATP consumption, the enigma concerning how RuvB translocates dsDNA needs to be elucidated. In conclusion, two flanking hexameric rings of RuvB of the RuvAB-Holliday-junction migration machinery translocate dsDNA unidirectionally.

## Conclusion and perspectives

When encountering a large scale of DNA damage attacks such as UV radiation or replication inhibitor antibiotics, bacteria activate the SOS response. Why *ruvAB* and *ftsK*, among the genes encoding many other ASCE DNA motor proteins, are induced during SOS seems puzzling. An insight into this mystery comes from DNA replication fork arrest resulting from a DNA damage attack that induces SOS. The lesion of the stalled forks generates the Holliday junction, necessitating RuvAB action to restore replication [95-98]. Similarly, terminal recombination intermediates resulting from chromosome replication must be resolved by FtsK. Intriguingly, the SOS repressors of several temperate phages also act in parallel with host LexA, inducing genes for viral DNA motors that lead to phage assembly and host cell lysis. This correlation hints at convergent evolution between the viral and the cellular DNA motors. While performing DNA translocation, phi29 gp16 ATPase and cellular FtsK use a revolution mechanism whereas RuvB seems to employ a rotation mechanism with different directionalities. The phage motor protein transports the viral genome unidirectionally by a check-valve mechanism [15,99] into the phage head (Figure 2A) whereas cellular FtsK resolves the duplicated chromosomes and translocates them bidirectionally from the septation region (Figure 2B). RuvB drives unidirectional migration of the Holliday junction. Each displays characteristic hexameric rings to encircle and pump dsDNA (Figure 2). This conserved strategy includes sequential subunit actions of ATP binding, DNA binding, ATP hydrolysis, and DNA translocation. DNA is translocated by a combination of chemical and mechanical reactions, albeit in different fashions. The phi29 gp16 ATPase uses a revolution mechanism of the entropy-DNA affinity switch between the subunits to generate a power stroke that pushes dsDNA inward without rotation of DNA and the pump. Cellular FtsK adopts a “rotary inchworm” mechanism of the hinged movement by α and β domains with sequential hand-on and hand-off events on dsDNA to effect transport without rotation. For RuvB, a mechanism differing to that used by gp16 was proposed, with dsDNA rotating by the same power stroke. Despite the significant progress in the nano-characterization of these DNA motors, the mechanism by which RuvB rotates and translocates dsDNA is still unclear, and the proposed mechanochemical-coupling mechanism is to be further tested at the nano-level. As the rotation mechanism causes coiling of DNA and incur a high ATP cost, future efforts should focus on these issues. It is plausible for cellular dsDNA motors to translocate dsDNA via revolution because such a mechanism does not cause topological stress on chromosome [100]. While the mechanisms of DNA translocation by phi29 gp16 ATPase and cellular FtsK have been extensively characterized *in vitro,* the future challenge is to validate these mechanisms *in vivo* by examining these DNA motors translocating DNA in live viruses and cells. Finally, SOS and the related ASCE motors may have profound implications. SOS can be induced by antibiotics not only via direct DNA damage but also via indirect and subsequent production of hydroxyl radicals [101,102] though they do not kill the bacteria [103]. SOS contributes to antibiotic-inducible bacterial biofilm formation [104-106] and vesiculation [107]. Moreover, convergent evolution has been proposed between SOS-inducible biofilm formation and tumor metastasis [106,108-111]. This convergence may allow bacteria under selective pressure of anti-cancer replication inhibitors to evolve anti-cancer phenotypes that may be facilitated by the SOS-related DNA motors [109-111]. Future study of these motors may provide insights into development novel anticancer therapy as well as anti-biofilm regimes.

## Abbreviations

dsDNA: Double stranded DNA; LexA: Locus for X-ray sensitivity A; RecA: Recombinase A; ssDNA: Single stranded DNA; ASCE: Additional Strand Catalytic E; AAA+: ATPases associated with a variety of cellular activities; *Lrp*: global response regulatory gene; EM: Electron microscopy; EcFtsK: FtsK of *E. coli*; FtsK_C_: C-terminal domain; PaFtsK: FtsK of *Pseudomonas aeruginosa.*

## Competing interests

None of the authors have financial or non-financial competing interests.

## Authors’ contributions

AW contributed by writing the section about RuvB. KP participated in writing the sections of SOS and FtsK and coordination of this project. TW conceived the design, synthesis and organization of this work as well as drafted the entire manuscript. All authors read and approved the final manuscript.

## Authors’ information

TW, PhD and MD, holds an Associate Professor position of Biology at Southwest Baptist University. His research is focused on the bacterial SOS response to DNA damaging antibiotics and the related physiology of biofilms, cell motility and vesiculation. KP, a senior pursuing a Biology major and Chemistry minor, is as an undergraduate researcher who has authored a research paper published in 2013. AW is a 2013-graduated *summa cum laude* with a Bachelor’s of Science in Biology and a concentration in Biomedical sciences.
